# Histomorphometric Evaluation of Subchronic and Chronic Effects of Novel Experimental Calcium Aluminate- and Calcium Silicate-Based Dental Cement Materials on Rat Liver, Kidney, Brain, and Spleen Tissues

**DOI:** 10.3390/jfb17050253

**Published:** 2026-05-20

**Authors:** Veljko Ilić, Sanja Milutinović-Smiljanić, Vladimir Biočanin, Jovana Kuzmanović Pfićer, Tatjana Tasić, Vesna Danilović, Nina Japundžić-Žigon, Smiljana Paraš, Vukoman Jokanović, Dejan Ćetković, Đorđe Antonijević

**Affiliations:** 1 School of Dental Medicine, University of Belgrade, 11000 Belgrade, Serbia; veljko.ilic@stomf.bg.ac.rs (V.I.); jovana.kuzmanovic@stomf.bg.ac.rs (J.K.P.); tatjana.tasic@stomf.bg.ac.rs (T.T.); vesna.danilovic@stomf.bg.ac.rs (V.D.); dejan.cetkovic@stomf.bg.ac.rs (D.Ć.); djordje.antonijevic@stomf.bg.ac.rs (Đ.A.); 2 Faculty of Dentistry in Pančevo, University of Business Academy, 21000 Novi Sad, Serbia; vladimirbiocanin@gmail.com; 3 Faculty of Medicine, University of Belgrade, 11000 Belgrade, Serbia; nina.zigon@med.bg.ac.rs; 4 Faculty of Natural Sciences and Mathematics, University of Banja Luka, 78000 Banja Luka, Bosnia and Herzegovina; smiljana.paras@pmf.unibl.org; 5 ALBOS d.o.o., 11000 Belgrade, Serbia; vukoman@vin.bg.ac.rs

**Keywords:** dental cement, long-term exposure, MTA, biocompatibility, in vivo model, calcium silicate cement, calcium aluminate cement

## Abstract

Although biocompatible calcium silicate cements (CSCs) and calcium aluminate cements (CACs) may induce local and systemic adverse effects. This study aimed to evaluate the subchronic and chronic effects of experimental CAC and CSC mixtures on rat liver, kidney, brain, and spleen tissue. Two experimental mixtures, CAC with added ZrO_2_ (ECCA + ZrO_2_) and CSC with added ZrO_2_ (ECCS + ZrO_2_), and mineral trioxide aggregate (MTA), were implanted intraalveolary in 36 male Wistar rats. Histomorphometry was conducted after 30 and 180 days on liver, kidney, brain, and spleen. Consistent results were observed in all material groups. Liver tissue inflammation ranged from none to minimal for all three materials. In kidney, ECCA + ZrO_2_ displayed a slightly better result than other two materials. In brain, after 180 days, both ECCA + ZrO_2_ and MTA showed a statistically significant reduction in perineural vacuolation (*p* < 0.05), and MTA showed a reduction in the percentage of intravascular congestion (*p* < 0.05). In spleen, a larger lymphoid follicle diameter was observed for ECCS + ZrO_2_ chronic group compared to other two materials (*p* < 0.05). ECCA + ZrO_2_, ECCS + ZrO_2_, and MTA caused none to minimal changes in liver, kidney, brain, and spleen following subchronic and chronic exposure.

## 1. Introduction

Calcium silicate cements (CSCs) are dental materials commonly used across various fields, including endodontics, restorative dentistry, and oral surgery. Since these materials come into close contact with vital pulp and periodontal tissue, they should exhibit high biocompatibility, non-toxicity, a non-carcinogenic nature, dimensional stability, and be easy to manipulate manually [1]. Although applied locally, some components of dental materials have the potential to enter circulation and affect distant organs [2]. Dissemination of metal ions, such as aluminum and bismuth [3,4,5] could consequently provoke systemic toxicity. Therefore, it is essential to consider the material’s characteristics to prevent undesirable effects, both locally and systemically.

The current “gold standard”, mineral trioxide aggregate (MTA), offers numerous advantages regarding its physicochemical and biological traits. It was proven to be sufficiently radiopaque, highly alkaline, minimally soluble, and exceedingly biocompatible [1,6,7]. However, it also has drawbacks related to its other physicochemical and biological properties. From the perspective of MTA’s biocompatibility, some components in its composition can induce local and systemic adverse effects [8]. For example, the bismuth oxide (Bi_2_O_3_) radiopacifier and heavy metals can cause undesirable tissue reactions. A study by Pelepenko et al. [5] showed that exposure to Bi_2_O_3_ from MTA negatively impacted cell viability and gene expression. Additionally, oxidative stress was observed in brain tissue following the application of CSC material [4]. Furthermore, a noticeable systemic reaction was reported in liver and kidney tissue after MTA implantation [9,10]. Prior studies suggested that replacing potentially cytotoxic Bi_2_O_3_ with inert zirconium dioxide (ZrO_2_) could enhance the overall biocompatibility of dental cement mixtures [11,12,13]. In light of these concerns, there is a clear need to identify a superior material with a different composition, incorporating an alternative radiopacifier.

For this purpose, calcium aluminate cements (CACs) are being studied, showing a significant potential for endodontic therapy appliances [14,15,16,17]. Our research team developed a new, highly refined CAC with satisfactory physicochemical properties, including a greater capacity for successful obturation, decreased micro- and nanoporosity, and fewer gaps between the cement material and the root canal walls [18]. It was also non-cytotoxic to the L929 and MRC-5 cell cultures and exhibited a superior elastic modulus [11,15,19]. Moreover, it proved to be biocompatible with liver and subcutaneous tissues, exhibiting higher compressive strength and a lower setting time compared to MTA [13,20]. However, additional studies are necessary to understand its biological effects fully.

Despite the large number of studies, the question of the effect of the CAC and CSC mixtures on internal organs remains. Most in vivo studies examined the influence of these materials on the subcutaneous tissue [13,17,21], dental pulp [22,23], and furcal perforations [7], but only a few assessed the short-term systemic effect of CACs [10,20]. However, these assessments did not include a long-term influence on internal organs and were primarily focused on liver and kidney tissue analysis.

Therefore, this in vivo study aimed to evaluate the subchronic and chronic effects of the experimental CAC and CSC mixtures enriched with ZrO_2_ on rat liver, kidney, brain, and spleen tissue, and to compare them with MTA (MTA+, Cerkamed, Stalowa Wola, Poland).

## 2. Materials and Methods

### 2.1. Experimental Cements and Specimen Preparation

The novel CAC was previously synthesised and described [15,18]. First, a pseudoboehmite sol (AlOOH) was prepared by dissolving aluminum tri-sec-butoxide (Sigma-Aldrich, St. Louis, MO, USA) in a 1:4 mixture of ethanol and water, with a molar ratio of 1:50. The mixture was heated to 85 °C for 2 h, cooled, and then sulfuric acid (Sigma-Aldrich, St. Louis, MO, USA) was added to achieve a molar ratio of 0.04. After reheating and refluxing for 1 h, the colloidal pseudoboehmite solution was obtained. This sol was mixed with stoichiometric CaCl_2_·5H_2_O (Merck, Darmstadt, Germany) and treated in an autoclave at 150 °C and 15 bar for 5 h. After cooling, the gel was dried at 150 °C, calcined at 1200 °C for 4 h, and then milled to achieve the desired particle sizes for the base CAC mixture. For the creation of the base CSC mixture, a commercial calcium silicate (−200 mesh, ≥90%, Sigma-Aldrich, St. Louis, MO, USA) was employed. Both base CAC and CSC mixtures were then enriched with 20 wt% of ZrO_2_ (Sigma-Aldrich, St. Louis, MO, USA), creating two experimental endodontic ceramics (EC): calcium aluminate cement with added zirconium dioxide (ECCA + ZrO_2_) and calcium silicate cement with added zirconium dioxide (ECCS + ZrO_2_). MTA (MTA+, Cerkamed, Stalowa Wola, Poland) was used as a control. The comprehensive characterization, regarding the surface morphology, elemental, chemical, and phase composition of these materials, was previously described [13,15,18].

Prior to implantation, the experimental cements were mixed with distilled water using a stainless-steel spatula on a glass slab, with a ratio of 1 g of powder to 0.33 mL of liquid. The MTA was prepared according to the manufacturer’s instructions. After mixing, all materials were inserted into sterile polyethylene tubes (10 mm long, with an inner diameter of 1 mm and an outer diameter of 1.5 mm) using a stainless-steel spatula and an endodontic compactor. The material was exposed at one end of the tube, resulting in an exposed surface area of approximately 0.785 mm^2^. The other tube end, oriented towards the oral cavity, was sealed with warm gutta-percha. Material-filled tubes were then allocated into three groups:

Group 1: tubes filled with ECCA + ZrO_2_ (*n* = 12)

Group 2: tubes filled with ECCS + ZrO_2_ (*n* = 12)

Group 3: tubes filled with MTA (*n* = 12)

### 2.2. Animals

All experiments of this animal study were performed according to the ARRIVE (Animal Research: Reporting of In Vivo Experiments) guidelines. Protocols for animal use were conducted and approved following the Directive of the European Parliament 2010/63/EU. The Ethics Committee of the School of Dental Medicine, University of Belgrade, grant no. 36/28 was issued on 7 July 2023.

In this study, 36 male rats (Rattus norvegicus albinus, Wistar), 9–10 weeks old, weighing approximately 300 g, with unlimited access to commercial food pellets and tap water, were maintained in a controlled environment (12 h/12 h light-dark cycle, temperature 22 ± 3 °C, and humidity 60 ± 10%) [24]. The weight of the rats was measured before implantation and prior to euthanasia. Cage-side observation included behavior patterns, changes in skin, fur, and mucous membranes, respiratory symptoms, motor activities, presence of convulsions, reflexes, ocular signs, cardiovascular signs, excessive salivation, piloerection, muscle tone, and gastrointestinal signs.

### 2.3. Experimental Design and Surgical Procedure

Rats were randomly allocated into three uniform groups (*n* = 12) according to the received material: ECCA + ZrO_2_, ECCS + ZrO_2_, or MTA. Animals from each material group were then divided into two subgroups (*n* = 6), according to the period of sacrifice (30 and 180 days). The surgical procedure was conducted under deep general anesthesia (ketamine (100 mg∙kg^−1^) and xylazine (10 mg∙kg^−1^), i.m. injection). The first upper right incisor was extracted from each animal using small upper anterior forceps. A material-filled tube was then inserted into the empty alveolar socket, and the extraction wound was sutured (Vicryl Plus 3-0, Ethicon, Raritan, NJ, USA) [3,25,26]. To prevent bacterial infection, gentamycin (Galenika AD, Belgrade, Serbia, 25 mg kg^−1^, i.m.) was administered for 3 days before surgery and on the day of the procedure. To reduce pain, rats were given metamizole (Novalgetol, Galenika AD, Belgrade, Serbia, 200 mg kg^−1^ day^−1^, i.m.) on the day of surgery and for the subsequent 2 days. Euthanasia was performed after 30 and 180 days using an overdose of thiopentone sodium (150 mg i.p.). After their administration, the loss of all reflexes was established, and the head was removed using a small animal decapitator guillotine. The biopsies were collected in a standardized order, beginning with the brain [27], followed by the liver, kidneys, and spleen [28]. This sequence was chosen to focus on organs most vulnerable to post-mortem autolysis, especially neural tissue, in order to reduce the chances of artifacts. At the same time, the first operator was tasked with the brain dissection, while the second handled the liver, kidneys, and spleen. The estimated time needed for collecting each organ was approximately: brain (~5 min), liver (2 min), kidneys (3 min for both), and spleen (2 min), totaling no more than 15 min for one animal. All organs were preserved in 10% neutral buffered formalin.

### 2.4. Histologic and Histomorphometric Analysis

Organ specimen tissue sections (4–5 μm thick) were stained with hematoxylin and eosin (H&E, Merck, Darmstadt, Germany), with every 5th section observed microscopically (Leitz LaborLux S Fluorescence Microscope, Ernst Leitz Wetzlar GMBH, Wetzlar, Germany). For each specimen, three non-overlapping microscopic fields (×40–×400) were captured with a digital camera (Leica DFC295, Wetzlar, Germany) and analysed using Leica Application Suite software (v4.5, Leica Microsystems, Wetzlar, Germany). The histomorphometric evaluation was conducted on the liver, kidney, brain, and spleen tissue. Two experienced examiners, blinded to the material groups and time intervals, assessed all histological images. The selection of measurement areas was obtained by automatised randomisation via Leica Application Suite software. Regions of interest containing artefacts and tissue overlaps were excluded.

#### 2.4.1. Liver

Two right medial lobe fragments of each liver were analysed. Liver tissue regions were sampled depending on the position relative to the liver vasculature: one area represented the peripheral region, second area represented the paracaval region, and third area represented the paraportal region. The peripheral region was defined as no more than 1 cm from the surface and periphery of right medial hepatic lobe. The paracaval region was located in the right medial lobe directly adjacent to the opening of the hepatic vein into the caudal vein cava. The paraportal (hilar) localization was immediately adjacent to the main branches of the portal vein followed from the hilum within anatomical lobe [29]. The measurement was performed in doubles. Additional Masson’s trichrome staining (MT, Merck, Darmstadt, Germany) was used for a more detailed analysis of liver fibrosis. The modified Histology Activity Index (HAI) was employed for the liver tissue assessment (Table 1) [30,31,32].

#### 2.4.2. Kidney

Cortical and medullary parts were assessed microscopically using the modified endothelial, glomerular, tubular, and interstitial tissue (EGTI) histology scoring system (Table 2) [33].

#### 2.4.3. Brain

The cerebral hemispheres from each animal were extracted, and sagittal sections were performed. Specimens included the hippocampus, and the region of interest was the *Cornu Ammonis* (CA), specifically the CA1 and CA2 areas. Observed parameters in brain tissue are presented in Table 3 [34,35,36]. To quantify the number of neurons in the hippocampus, nuclei larger than 20 μm^2^ were included. Smaller nuclei were considered to be from glial cells and were excluded [37].

#### 2.4.4. Spleen

One fragment of each animal’s spleen was submitted to semi-serial sections. Observed parameters are presented in Table 4 [38,39].

### 2.5. Statistical Analysis

Statistical analyses were conducted using SPSS version 29.0 (IBM Corp., Armonk, NY, USA). The normality of the data was assessed with the Kolmogorov-Smirnov test. Since all data were found to be non-normally distributed, the Friedman test was used for within-group analysis, while the Kruskal-Wallis test was applied for between-group comparisons. Post hoc analysis was performed using the Bonferroni test. A significance level of *p* < 0.05 was set to determine statistical significance. The values of brain and spleen tissue analysis were presented as median with interquartile range (IQR). For the liver and kidney tissue assessment, the cumulative HAI and EGTI scores were calculated by summing the mean values of individual observed parameters (Table 1 and Table 2), followed by descriptive interpretation [30,31,32].

## 3. Results

During the experiment, the rats’ weight did not vary more than 10%, and all animals were without behavioural and clinical abnormalities of the skin, fur, mucous membrane changes, respiratory symptoms, pathological motor and neurological activities, ocular, cardiovascular, and gastrointestinal signs, excessive salivation, piloerection, and muscle tone.

### 3.1. Liver

**Figure 1:**
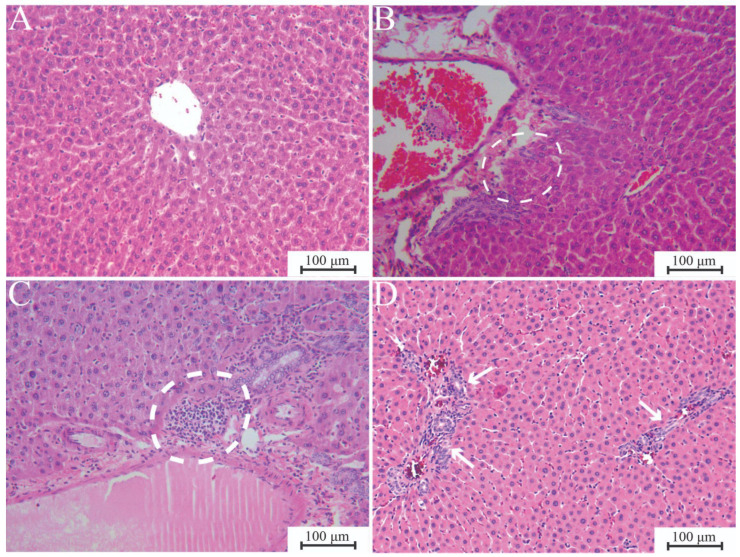
Representative photomicrographs of liver histological cross-sections. (**A**) Liver tissue with no observable pathological changes; MTA 180 days, H&E ×200. (**B**) Mild periportal piecemeal necrosis of hepatocytes (dashed circle). Inflammatory cells are present in small numbers, permeating the liver parenchyma from the portal tracts, leading to localised patchy destruction of individual hepatocytes. The inflammation is spreading from the portal tract into the surrounding liver tissue, partially destroying the “limiting plate” (a cell layer surrounding the portal tract) and resulting in a localised, irregular, fragmented cell pattern; ECCA + ZrO_2_, 30 days, H&E ×200. (**C**) Portal triad with an inflammatory cell infiltrate (dashed circle); MTA 30 days, H&E ×200. (**D**) Increased fibrous tissue (arrows), slightly more pronounced compared to the normal histological appearance; ECCS + ZrO_2_ 180 days, MT ×200. ECCA + ZrO_2_, experimental calcium aluminate cement with added zirconium dioxide; ECCS + ZrO_2_, experimental calcium silicate cement with added zirconium dioxide; MTA, mineral trioxide aggregate.

The liver tissue changes are summarised in Table 5, with representative photomicrographs displayed in Figure 1. Consistent results were observed across all material groups during the same period, ranging from no to minimal histological changes. Subchronic exposure induced a slightly more pronounced tissue reaction compared to chronic exposure. The ECCS + ZrO_2_ exhibited marginally better results during the subchronic exposure, while the chronic tissue reaction of MTA appeared to be somewhat milder than that of the other two materials.

### 3.2. Kidney

**Figure 2:**
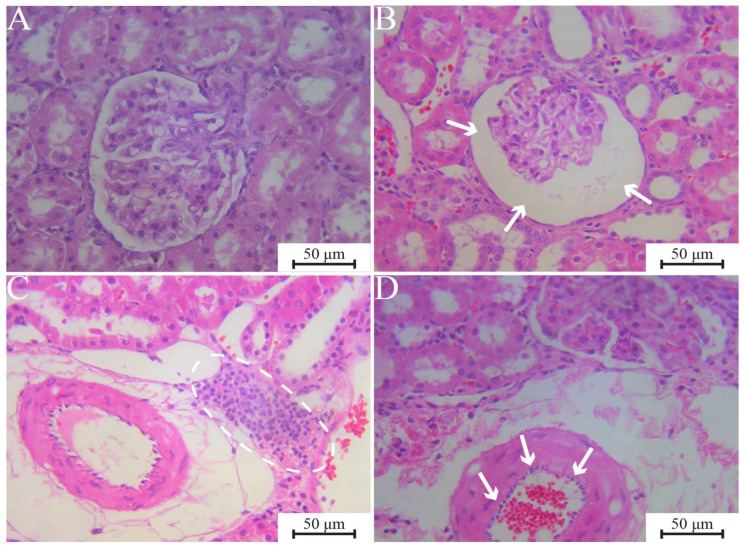
Representative photomicrographs of kidney histological cross-sections (H&E). (**A**) Renal tissue with preserved glomerular and tubular architecture; ECCA + ZrO_2_ 180 days, ×400. (**B**) Glomerular capillary tuft showing mild retraction and partial collapse without evident basement membrane wrinkling (arrows); ECCS + ZrO_2_ 30 days, ×400. (**C**) Interstitial inflammatory infiltrate (dashed circles) observed around blood vessels; MTA 30 days, ×200. (**D**) Endothelial swelling of an arterial blood vessel (arrows), resulting in thickening of the vascular wall; ECCA + ZrO_2_ 30 days, ×400. ECCA + ZrO_2_, experimental calcium aluminate cement with added zirconium dioxide; ECCS + ZrO_2_, experimental calcium silicate cement with added zirconium dioxide; MTA, mineral trioxide aggregate.

The EGTI scores are presented in Table 6, with the corresponding photomicrographs displayed in Figure 2. The EGTI score for ECCA + ZrO_2_ was evaluated as none during both the subchronic and chronic exposure periods. ECCS + ZrO_2_ and MTA yielded similar findings; however, the chronic impact of MTA was also reported as none, parallel to the results for ECCA + ZrO_2_.

### 3.3. Brain

**Figure 3:**
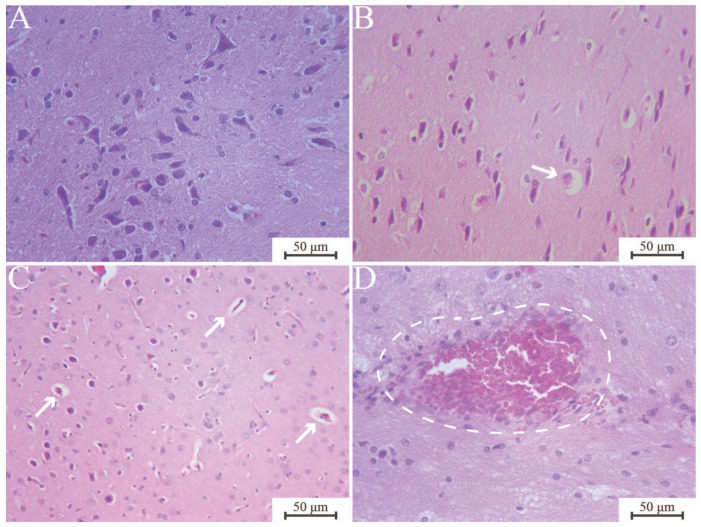
Representative photomicrographs of brain histological cross-sections (H&E). (**A**) Brain tissue with normal histological appearance; MTA 30 days, ×400. (**B**) Necrotic neuron (arrow) exhibiting eosinophilic cytoplasm and nuclear shrinkage (“red dead” neuron) ECCS + ZrO_2_ 30 days, ×400. (**C**) Neurons exhibiting perinuclear vacuolization (arrows) with small vacuoles, appearing as empty spaces, encircle the neurons; MTA 180 days, ×400. (**D**) Congested blood vessel (dashed circle) with lumen filled with erythrocytes; ECCA + ZrO_2_ 180 days, ×400. ECCA + ZrO_2_, experimental calcium aluminate cement with added zirconium dioxide; ECCS + ZrO_2_, experimental calcium silicate cement with added zirconium dioxide; MTA, mineral trioxide aggregate.

The brain tissue analysis values are presented in Table 7, with the corresponding photomicrographs displayed in Figure 3. Histomorphometric evaluation showed that the contribution of neurons with abnormalities to the total number of neurons is negligible. The percentage of neurons with perineural vacuolation decreased significantly in the chronic compared to the subchronic interval for both ECCA + ZrO_2_ and MTA (*p* < 0.05). The presence of necrotic neurons was low at both periods, without statistical significance among the tested materials (*p* > 0.05). MTA showed a reduction in the percentage of intravascular congestion after 180 days (*p* < 0.05).

### 3.4. Spleen

**Figure 4:**
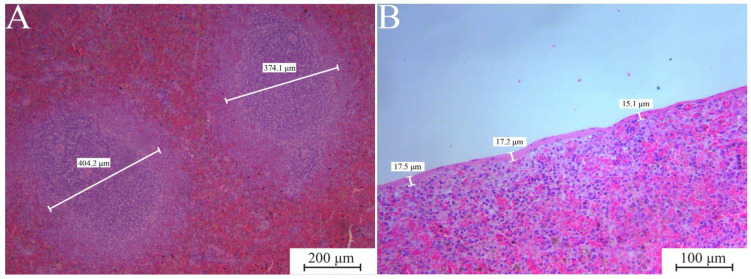
Representative photomicrographs of spleen histological cross-sections (H&E). (**A**) Measurement of lymphoid follicle diameters, ECCS + ZrO_2_ 180 days, ×100. (**B**) Measurement of splenic capsule thicknesses, MTA 30 days, ×200. ECCS + ZrO_2_, experimental calcium silicate cement with added zirconium dioxide; MTA, mineral trioxide aggregate.

Structural changes, abnormal cells, inflammation, hypertrophy, hyperplasia of the red and white pulp, and parenchymal fibrosis were either absent or present in less than 20% of the spleen tissue in all histological slides. The results regarding the diameters of the capsule and lymphoid follicles are presented in Table 8. A statistically significant difference was observed only in the lymphoid follicle diameter of the ECCS + ZrO_2_ chronic group (*p* > 0.05). Representative photomicrographs illustrating the thickness of the capsule and the diameter of the lymphoid follicles are shown in Figure 4.

## 4. Discussion

Calcium silicate-based materials are known to release heavy metals into surrounding tissues [3]. These substances can enter the bloodstream and spread to distant organs, including the liver, kidneys, brain, and spleen. Therefore, it is recommended to evaluate the systemic toxicity of new dental materials [40], which justifies the aim of this study. The liver, kidneys, brain, and spleen are considered critical organs for assessing potential treatment-related lesions [9,10,21,33,34,36,38,39]. However, studies detailing their chronic effects are extremely rare. Additionally, no studies have simultaneously examined the effects of dental cements on all four organs within the same experiment. Furthermore, histological assessments of brain and spleen tissues following dental cement implantation are rarely reported in the literature. The lack of cardiac and pulmonary evaluation may represent a limitation of this study, as these organs are among the first to be affected [41]. Future studies should include these organs to better assess the early systemic distribution of material components.

In vivo models are regarded as the most authoritative method for evaluating the biocompatibility of dental materials, as they simulate the interaction between materials and tissues. Before selecting an appropriate in vivo model, it is essential to conduct extensive in vitro studies on cell lines [42]. Our preliminary studies on the L929 and MRC-5 cell lines [11,15,19] demonstrated promising results regarding the cytotoxicity and genotoxicity of CAC and CSC experimental cements. These findings further support the use of animal models in our current investigations.

Wistar rats were chosen for this study due to their ease of maintenance, genetic uniformity, adaptability to laboratory conditions, and their anatomical, genetic, and physiological similarities to humans [43,44]. We selected the minimum number of experimental animals necessary to yield meaningful data while adhering to the 3Rs principle and ethical guidelines. The number of experimental animals and samples required was determined based on ISO standard recommendations, relevant biological studies, and statistical analyses [21,24,45,46]. This approach not only reduces the total number of animals used in research but also enhances the efficiency and cost-effectiveness of scientific investigations [47]. While the absence of a negative control group (non-implanted rats) may be a limiting factor, this omission can be justified by the understanding that certain tissue changes will inevitably occur following the implantation of dental cement. Additionally, the lack of negative control can be viewed as an ethical advantage, as it spared the sacrifice of an additional 12 rats. However, a limitation of this study is the absence of a dedicated control group. While comparisons with negative control groups from previously published studies were considered, substantial heterogeneity in experimental design, animal characteristics, implantation protocols, and histological evaluation methods precluded meaningful and unbiased direct comparison. The absence of a dedicated negative control group in present study may limit the interpretation of subtle histological changes and prevent definitive comparison with baseline physiological tissue morphology. Future studies should include appropriate negative control groups at multiple time points to enable more robust interpretation and clearer comparison between implanted and untreated animals.

Although MTA is considered the “gold standard”, extensively studied and widely used as a clinically relevant commercial reference and control material for calcium silicate–based cements, CACs represent a distinct class of biomaterials with different physicochemical characteristics. Therefore, inclusion of a commercially available calcium aluminate-based control material could have provided a more comprehensive comparative analysis with the experimental ECCA + ZrO_2_ formulation.

Studies by Demirkaya et al. [3,4] represent the most comparable models to the present study, as they were conducted on Wistar rats and involved in vivo exposure to CSC dental materials. Demirkaya et al. utilised implantation into the extraction socket of the upper incisor, although differences in animal age and study design were noted. These studies primarily focused on the quantification of elemental release (e.g., aluminum levels in brain, kidney, and blood plasma), rather than histological evaluation. Their findings demonstrated measurable systemic distribution of material components, while negative control groups exhibited lower baseline values, supporting the concept that implantation of foreign materials may induce detectable systemic changes. In contrast, several studies using Wistar rats applied different experimental approaches, such as subcutaneous implantation [10,13,21] or direct application to exposed dental pulp [23]. Although these studies provide valuable information regarding the biological behavior and safety profile of similar materials, their experimental conditions differ from the present study and therefore limit direct comparability. Additionally, other investigations employed different animal models, including Sprague–Dawley rats [48] and dogs [22], or alternative exposure routes such as oral administration [49]. Variations in species, implantation site, and exposure conditions may significantly influence material behavior, systemic distribution, and metabolic response. Consequently, these studies should be interpreted as supportive but indirect evidence of material safety, rather than directly comparable models. While existing literature consistently supports the favorable biocompatibility of CAC and CSC materials across different models and species, the differences in experimental design must be carefully considered when interpreting and comparing results.

Endodontic therapy should, preferably, provide lifelong results. Thus, applied dental cements should induce a multi-decade favorable outcome and ideally remain stable and biocompatible within the oral tissues. The euthanasia time points in our study were selected according to standards describing the subchronic (30 days) and chronic (180 days) inflammation periods [45]. The 180-day implantation period in rats is especially important since it is roughly equivalent to 18 years in humans [50]. Therefore, the methodology of our study mimicked the long-term effects of endodontic therapy on internal organs. However, studies investigating the impact of these materials beyond 6 months after the implantation are very scarce. This fact highlights the importance, but also complicates the comparison of our results with other studies.

The intraalveolar implantation model emphasises the advantage of direct contact between the material and periodontal ligament, which is crucial for wound healing and new tissue formation [25]. Periodontal tissue, rich in blood vessels, facilitates the systemic dissemination of material components through the bloodstream, creating a suitable environment for investigating their influence on distant organs [3,4,25]. This method allows for the more precise simulation of root-end apical surgery and perforation management procedures. Consequently, we considered the intraalveolar implantation methodology more authoritative than the subcutaneous implantation, as it better replicates the clinical conditions of material application. However, there is a lack of research investigating the systemic influence of dental CAC, particularly when implanted directly into the tooth alveolus.

Some differences in systemic responses observed after both intraalveolar and subcutaneous implantation can be explained by the distinct biological environments and absorption pathways associated with these tissues. While systemic diffusion of degradation products cannot be fully ruled out in either model, subcutaneous implantation represents a highly vascular route, without anatomical barriers, that favors faster absorption and systemic dissemination of ions or particulates, as documented in ISO 7405-based models [25,51]. For this reason, subcutaneous implantation method is commonly utilised in material studies and is recommended by the ISO standard [52]. In contrast, alveolar implantation involves bone and periodontal tissues with slower fluid turnover and sequential osteogenic phases, limiting and delaying systemic absorption [53]. Therefore, differences in vascularity, tissue density, and healing dynamics likely account for the slower and more localized resorption observed in the oral site, potentially explaining the minimal histological changes observed through organs in this study. While subcutaneous implantation models are useful for assessing the maximum potential for systemic exposure, intraalveolar implantation model may better reflect clinically relevant conditions, where absorption is often more restricted and influenced by local tissue characteristics.

Implanted CAC and CSC materials undergo physicochemical interactions with surrounding tissues, resulting in the release of calcium ions, hydroxyl ions, heavy metals, and trace elements from their composition [5]. While these processes contribute to their bioactivity and biomineralization, they may also facilitate the migration of material-derived components beyond the implantation site. Previous studies have shown that these released components can enter systemic circulation. Demirkaya et al. [3,4] and Karunakaran et al. [8] have reported measurable accumulation of elements such as aluminum and nanoparticles in distant organs, including the brain, kidneys, and blood plasma, following in vivo exposure, indicating systemic dissemination. Nanoparticulated materials are specifically prone to this issue as they can easily enter the bloodstream [8]. Our previous study identified that ECCA contains particles ranging in size from 15 nm to 425 nm [13], categorising it as a nanomaterial. In addition, studies have shown that MTA exhibits continuous ion release and surface degradation in biological fluids, facilitating their interaction with surrounding tissues and potentially their transport via the bloodstream. Heavy metals in MTA’s composition, such as arsenic ions [54,55] and Bi_2_O_3_ [5], may induce systemic toxicity. When arsenic ions come into contact with blood, they are transported by erythrocytes and can accumulate in the liver, kidneys, central nervous system, and other internal organs [56]. Furthermore, Demirkaya et al. [3] found elevated levels of aluminum in the blood plasma and liver of rats implanted with CSCs, suggesting that aluminum is released from these materials. The degree of organ damage can vary based on the type of material used, the dosage, and the duration of exposure. Another potential cause of systemic side effects is the production of cytokines, such as IL-1 and IL-6, at the implantation site, which can be transported to the liver and other distant organs [57,58]. It is reasonable to expect systemic disturbances following the application of endodontic materials, and the abovementioned pathways should be considered when interpreting the biological responses observed in distant organs. Although direct tracking of the materials’ components was not conducted in this study, the observed systemic effects may be explained by the previously mentioned mechanisms.

The examination of the liver, an organ crucial for detoxifying harmful substances, aligns with numerous studies that analyse the effects of potentially toxic materials on the body using histomorphometry [30,31,32]. In our study, the HAI of the tested materials was assessed as non-existent or minimal across all experimental groups (Table 5). Similar findings were reported by Delfino et al. [21], where three dental CSCs and CACs did not cause morphological changes in liver tissue and demonstrated bioactive potential. However, several studies indicated a more pronounced inflammatory response in liver and kidney tissues [9,10] following the application of similar materials. Garcia et al. [10] found that both MTA and CAC induced changes in liver and kidney tissues, with MTA showing lesser effects, albeit these effects diminished over time. The discrepancies in the literature may be attributed to differences in methodology, observation periods, and observed tissue parameters. Our results indicate that MTA exhibited a slightly higher HAI score after 30 days (2.0) compared to ECs (1.8 and 1.4, respectively). After 180 days, HAI scores were lower in all material groups, with CSCs demonstrating a somewhat better outcome (Table 5). Nevertheless, the overall results of the liver tissue analysis can be deemed satisfactory, indicating favorable biocompatibility for all three materials over both time frames.

Pathological changes in blood-filtering organs may occur after the implantation of dental materials, with previous studies noting the morphological changes in the kidneys as systemic effects linked to subcutaneous [9,10] and intramuscular [48] implantation of calcium silicate-based materials. In our study, however, we observed that pathological changes in the endothelial, glomerular, tubular, and interstitial tissues were either absent or minimal (Table 6). The ECCA + ZrO_2_ mixture had an EGTI score of 0 at both 30 and 180 days, while the other materials had varying scores: ECCS + ZrO_2_ recorded 2.1 at 30 days and 0.6 at 180 days, and MTA recorded 2.3 at 30 days and 0 at 180 days. Considering that the EGTI score can reach a maximum of 14 points, we find our scores of tested materials to be encouraging. These results indicate a slightly favorable effect of ECCA + ZrO_2_ on kidney tissue.

The impact of dental materials on brain tissue morphology has not been extensively studied, particularly over long periods. This study is the first to investigate this issue in detail. Previous research has shown that certain small particles and active substances can cross the blood-brain barrier [53]. Therefore, it is reasonable to assume that some components of endodontic cements may behave similarly. Our focus on the hippocampus aligns with earlier work by Zulfiqar [59], which identified the hippocampus as a primary site for neurotoxicity. Furthermore, selecting the CA1 and CA2 regions of the hippocampus is consistent with prior studies that have explored the pharmacological effects of certain drugs and materials, as well as social memory, in these areas [37,60]. Based on the analysis of brain tissue, both the ECCA + ZrO_2_ and MTA at 180 days showed a significant reduction in neurons with perineural vacuolation compared to the 30-day period. It is well known that vacuoles can develop spontaneously within and around neurons, particularly in ageing animals. These vacuoles are non-specific and do not necessarily indicate toxic effects; they may also arise from trauma or prolonged storage of tissues in 70% alcohol [61]. However, our experiments were conducted on strictly controlled young animals, and the tissue was carefully processed histologically. Therefore, we believe the observed neuronal vacuolization may result from toxic components in the dental mixture. Research has shown that type 3 cell death initiates with the swelling of intracellular organelles, followed by the formation of cytoplasmic spaces that can merge. The plasma membrane and cell membrane integrity are gradually compromised, which is associated with necrosis [62]. In our study, we observed necrotic neurons sporadically across all materials. The limited number of affected neurons and the minimal extent of neural tissue involvement, along with the absence of the Fluoro-Jade technique to distinguish necrotic neurons from artefacts more precisely, suggest that the observed pathological changes may not have significant clinical relevance. Furthermore, the interstitial hemorrhage observed in one specimen was likely caused artificially during the extraction of the rat’s brain and was not included in the statistical analysis. Although the results are promising, we remain concerned about aluminum levels in brain tissue, especially since Demirkaya et al. [4] reported higher aluminum levels and oxidative stress in brain tissue induced by implanted calcium silicate-based materials. Given that our ECCA + ZrO_2_ mixture contains high aluminum content, future studies examining the accumulation of aluminum in brain tissue would be valuable. Literature on the effects of dental cement implantation on brain tissue is limited, so we were unable to compare our findings with previous research.

Lymphoid organs, such as the spleen, are sensitive to various exponents due to the filtering of blood and lymph. However, we did not find any literature reference describing the effect of dental cements on the spleen after the intraalveolar application. In this paper, both subchronic and chronic exposure resulted in the spleens of all tested animals displaying typical histological features. Each spleen exhibited a surrounding capsule with trabeculae extending into the parenchyma, which consisted of distinct lymphoid follicles within the white pulp and red pulp (Figure 4). We did not notice a statistically significant difference in spleen capsule thickness between material groups and periods. Our previous results of the analysed spleen tissue after the oral administration of dental cement suspension did not show any pathological changes [49], which is in accordance with the current findings. Lymphoid follicles in the spleen play a key role in the adaptive immune response [63]. When foreign substances enter the body, B cells are activated, leading to several outcomes: (1) B cells multiply in response to the antigen, causing lymphoid follicles to enlarge; (2) formation of germinal centers where B cells rapidly divide and differentiate into plasma and memory B cells; (3) immune cells release inflammatory cytokines that recruit and activate more immune cells. If the implanted material stays in the tissue, it can lead to chronic inflammation, resulting in ongoing B cell activation and a prolonged immune reaction [63,64]. Following this statement, dental cement could induce chronic inflammation. However, we did not observe a difference in the size of the lymphoid follicles after the subchronic and chronic exposure, assuming the implanted materials did not cause chronic irritation and excessive stimulation. Although after 180 days ECCS + ZrO_2_ had significantly larger follicles (~385 μm) than the other two materials, it did not exceed the diameter of 600 ± 300 µm, described as physiological by Santana et al. [65].

The general lack of a standardised methodology for the examination of dental materials and their effect on internal organs presented an issue for the analysis of the data obtained, making it harder to adapt the methodology used in this study. To ease the examination and results comparison, it would be desirable to define a uniform protocol for future dental material toxicity research.

We consider the lack of our study to be the absence of immunohistochemical staining, which would allow us to determine pathological changes more accurately. Also, additional blood analysis might have been beneficial to further evaluate the serum levels of alanine aminotransferase, aspartate aminotransferase, urea, and creatinine to assess liver and kidney functions, as presented in literature [9,10]. Future studies should include molecular analyses, particularly the quantification of pro-inflammatory cytokines such as IL-1β, IL-6, and TNF-α, to gain deeper insight into inflammatory mechanisms.

Modern dentistry emphasises the preservation of natural tooth function and the postponement of implant or prosthodontic therapy, whenever possible. Considering that materials used in endodontic treatment should support these principles, the favorable properties of CAC and CSC indicate their potential applicability in routine dental practice. In our study, a long-term exposure to CAC and CSC experimental materials has proven to be relatively safe, with cements not causing significant histological changes in the organs; however, there were still some minimal alterations observed. We believe that for healthy patients without systemic diseases affecting specific organs, endodontic therapy with the use of these materials would generally be safe. Nevertheless, dental practitioners should exercise caution when applying these materials in patients with already present liver, kidney, brain, or spleen damage, as the additional irritation from the implanted material could exacerbate the altered tissues. This assumption could serve as a goal for future research, where experimental dental cements might be applied in an animal model with induced chronic systemic diseases to examine their effects on already altered tissue.

## 5. Conclusions

This study demonstrates, for the first time, the systemic histological response following subchronic and chronic exposure to calcium silicate- and calcium aluminate-based dental materials. Under the conditions of the present study, both experimental and commercial (MTA+) mixtures showed comparable histological findings in the liver, kidney, brain, and spleen tissues, without evidence of major pathological alterations. These findings suggest a generally favorable systemic biocompatibility profile of the tested materials within the limitations of the experimental design. Furthermore, given that the assessment was limited to histological evaluation, the possibility of subtle, molecular, or subcellular biological effects that are not detectable by this method cannot be excluded. Although this study provides valuable preliminary data regarding the systemic effects of calcium aluminate- and calcium silicate-based dental cements, future studies incorporating advanced analytical techniques are necessary to further clarify the toxicological implications of these materials.

## Figures and Tables

**Table 1:** Modified Histology Activity Index (HAI) ^a^ used for the evaluation of liver tissue.

	Scoring
Periportal ± bridging necrosis (A)	(0) None; (1) Mild piecemeal necrosis; (3) Moderate piecemeal necrosis (involves less than 50% of the circumference of most portal tracts); (4) Marked piecemeal necrosis (involves more than 50% of the circumference of most portal tracts); (5) Moderate piecemeal necrosis plus bridging necrosis ^d^; (6) Marked piecemeal necrosis plus bridging necrosis ^d^; (10) Multilobular necrosis ^e^
Intralobular degeneration and focal necrosis ^b^ (B)	(0) None; (1) Mild (acidophilic bodies, ballooning degeneration, and scattered foci of hepatocellular necrosis <⅓ of lobules of nodule, nuclei pyknosis, arterial dilatation); (3) Moderate (involvement of ⅓–⅔ of lobules or nodules); (4) Marked (involvement of > ⅔ of lobules or nodules)
Portal inflammation (C)	(0) None; (1) Mild (sprinkling of inflammatory cells in less than ⅓ of portal tracts; (3) Moderate (increased inflammatory cells in ⅓–⅔ of portal tracts; (4) Marked (dense packing of inflammatory cells in more than ⅔ of portal tracts
Fibrosis (D)	(0) None; (1) Fibrous expansion; (2) Bridging fibrosis; (3) Cirrhosis ^c^
HAI interpretation (A+B+C+D)	(0) None; (1–4) Minimal; (5–8) Mild; (9–12) Moderate; (13–21) Marked tissue changes

^a^ HAI score is the combined score for necrosis, inflammation, and fibrosis. ^b^ Degeneration-acidophilic bodies, ballooning; focal necrosis-scattered foci of hepatocellular necrosis. ^c^ Loss of normal hepatic lobular architecture with fibrous septae separating and surrounding nodules. ^d^ Bridging is defined as ≥2 bridges in the liver biopsy specimen; no distinction is made between portal-portal and portal-central linkage. ^e^ Two or more contiguous lobules with panlobular necrosis.

**Table 2:** Modified EGTI histology scoring system used for the evaluation of kidney tissue.

	Scoring
Endothelial tissue (A)	(0) No damage; (1) Endothelial swelling; (2) Endothelial disruption; (3) Endothelial loss
Glomerular tissue (B)	(0) No damage; (1) Thickening of the Bowman capsule; (2) Glomerular tuft retraction; (3) Glomerular fibrosis
Tubular tissue (C)	(0) No damage; (1) Loss of brush border in <25% of tubular cells and preserved integrity of basal membrane; (2) Loss of brush border in >25% of tubular cells and thickened basal membrane; (3) (Plus) inflammation, cast formation, and necrosis in <60% of tubular cells; (4) (Plus) necrosis present in >60% of tubular cells
Interstitial tissue (D)	(0) No damage; (1) Inflammation and hemorrhage in <25% of tissue; (2) (Plus) necrosis in <25% of tissue; (3) Necrosis in 25–60% of tissue; (4) Necrosis in >60% of tissue
EGTI score interpretation (A+B+C+D)	(0) None; (1–4) Minimal; (5–8) Moderate; (9–14) Severe pathological changes

**Table 3:** Histological parameters evaluated in brain tissue.

The percentages of neurons manifesting perineuronal vacuolation and neuronal necrosis, regarding the total number of neurons per field of view
The percentages of the intracerebral hemorrhage, regarding the total area of the field of view
The percentage of the intracerebral blood vessels congestion, regarding the total blood vessel area

**Table 4:** Histological parameters evaluated in spleen tissue.

Inflammation; Hypertrophy of the red and white pulp; Hyperplasia of the red and white pulp; Parenchymal fibrosis	Score
	(0) Absent, or less than 20%; (1) Slight, 20–30%; (2) Moderate, 31–60%; (3) Severe, present in more than 60% of spleen tissue
Capsule thickness and lymphoid follicle diameter (μm)	

**Table 5:** Histological evaluation scores of liver tissue across experimental groups.

	ECCA + ZrO_2_		ECCS + ZrO_2_		MTA	
	30 Days	180 Days	30 Days	180 Days	30 Days	180 Days
Periportal and bridging necrosis	0.4 ± 0.5	0.3 ± 0.5	0.2 ± 0.4	0.1 ± 0.4	0.2 ± 0.4	0.2 ± 0.4
Intralobular degeneration and focal necrosis	0	0	0	0	0	0
Portal inflammation	0.4 ± 0.5	0.3 ± 0.5	0.2 ± 0.4	0	1.0 ± 0.0	0
Fibrosis	1.0 ± 0.0	1.0 ± 0.0	1.0 ± 0.0	0.4 ± 0.5	0.8 ± 0.4	0.2 ± 0.4
Histology Activity Index score (interpretation)	1.8 (minimal tissue changes)	1.6 (minimal tissue changes)	1.4 (minimal tissue changes)	0.5 (no tissue changes)	2.0 (minimal tissue changes)	0.4 (no tissue changes)

ECCA + ZrO_2_, experimental calcium aluminate cement with added zirconium dioxide; ECCS + ZrO_2_, experimental calcium silicate cement with added zirconium dioxide; MTA, mineral trioxide aggregate.

**Table 6:** Histological evaluation scores of kidney tissue across experimental groups.

	ECCA + ZrO_2_		ECCS + ZrO_2_		MTA	
	30 Days	180 Days	30 Days	180 Days	30 Days	180 Days
Endothelial tissue	0	0	0	0	0.5 ± 0.3	0
Glomerular tissue	0	0	1.8 ± 0.3	0	0.9 ± 0.4	0
Tubular tissue	0	0	0	0	0.1 ± 0.1	0
Interstitial tissue	0	0	0.3 ± 0.2	0.6 ± 0.2	0.8 ± 0.2	0
EGTI score (interpretation of pathological tissue changes)	0 (None)	0 (None)	2.1 (Minimal)	0.6 (Minimal)	2.3 (Minimal)	0 (None)

ECCA + ZrO_2_, experimental calcium aluminate cement with added zirconium dioxide; ECCS + ZrO_2_, experimental calcium silicate cement with added zirconium dioxide; MTA, mineral trioxide aggregate.

**Table 7:** Observed histopathological findings in brain tissue across experimental groups.

	ECCA + ZrO_2_		ECCS + ZrO_2_		MTA	
	30 Days	180 Days	30 Days	180 Days	30 Days	180 Days
Perineuronal vacuolation	12.6 (9.4–16.4)	4.5 (2.4–7) ^#^	13 (7.4–15.8)	4.3 (2.7–11.2)	11.6 (7.6–22)	6.1 (5.6–7.5) ^#^
Neuronal necrosis	4.3 (2.8–10)	1.6 (0.9–4.1)	3.2 (2.2–5.1)	0.4 (0–2.2)	4.4 (3.2–7.7)	4.6 (3.2–5.2)
Intracerebral hemorrhage	0 (0–0)	0 (0–0)	0 (0–0)	0 (0–0)	0 (0–0)	0 (0–0)
Intracerebral blood vessel congestion	33.7 (23–34.1)	4.8 (0.7–23.8)	16.7 (10–28.7)	7.1 (4.2–10.1)	23.3 (14.8–46)	7.1 (4–11.1) ^#^

ECCA + ZrO_2_, experimental calcium aluminate cement with added zirconium dioxide; ECCS + ZrO_2_, experimental calcium silicate cement with added zirconium dioxide; MTA, mineral trioxide aggregate. ^#^ vs. 30 days, Friedman test (*p* < 0.05).

**Table 8:** Observed histopathological findings in spleen tissue across experimental groups.

	ECCA + ZrO_2_		ECCS + ZrO_2_		MTA	
	30 Days	180 Days	30 Days	180 Days	30 Days	180 Days
Inflammation	0 (0–0)	0 (0–0)	0 (0–0)	0 (0–0)	0 (0–0)	0 (0–0)
Hypertrophy of the red and white pulp	0 (0–0)	0 (0–0)	0 (0–0)	0 (0–0)	0 (0–0)	0 (0–0)
Hyperplasia of the red and white pulp	0 (0–0)	0 (0–0)	0 (0–0)	0 (0–0)	0 (0–0)	0 (0–0)
Parenchymal fibrosis	0 (0–0)	0 (0–0)	0 (0–0)	0 (0–0)	0 (0–0)	0 (0–0)
Capsule thickness (μm)	22.7 (18.1–24.7)	19.5 (18.2–20.5)	17 (15.4–19.3)	15.1 (14–18.8)	18.5 (16–20.2)	18.7 (14.3–20.5)
Lymphoid follicle diameter (μm)	303.1 (280–335.3)	294.5 (267–344.5)	324.2 (267.2–383)	385.4 (374.1–412.5) *	330.1 (301.6–360.7)	323.4 (271.4–372.1)

ECCA + ZrO_2_, experimental calcium aluminate cement with added zirconium dioxide; ECCS + ZrO_2_, experimental calcium silicate cement with added zirconium dioxide; MTA, mineral trioxide aggregate. * vs. ECCA + ZrO_2_ and MTA, Kruskal-Wallis test (*p* < 0.05).

## Data Availability

The data presented in this study are available on request from the corresponding author due to privacy, legal, or ethical reasons.

## Abbreviations

CSC	Calcium silicate cement
CAC	Calcium aluminate cement
ECCA + ZrO_2_	Calcium aluminate cement with added zirconium dioxide
ECCS + ZrO_2_	Calcium silicate cement with added zirconium dioxide
MTA	Mineral trioxide aggregate
EC	Endodontic ceramic
HAI	Histology activity index
EGTI	Endothelial, glomerular, tubular, and interstitial tissue
CA	Cornu Ammonis

## References

[1] Parirokh M., Torabinejad M. (2010). Mineral trioxide aggregate: A comprehensive literature review—Part I: Chemical, physical, and antibacterial properties. J. Endod..

[2] Stanley H.R. (1993). Effects of dental restorative materials: Local and systemic responses reviewed. J. Am. Dent. Assoc..

[3] Demirkaya K., Demirdöğen B.C., Torun Z.Ö., Erdem O., Çetinkaya S., Akay C. (2016). In vivo evaluation of the effects of hydraulic calcium silicate dental cements on plasma and liver aluminium levels in rats. Eur. J. Oral Sci..

[4] Demirkaya K., Demirdöğen B.C., Torun Z.Ö., Erdem O., Çırak E., Tunca Y. (2017). Brain aluminium accumulation and oxidative stress in the presence of calcium silicate dental cements. Hum. Exp. Toxicol..

[5] Pelepenko L.E., Marciano M.A., Shelton R.M., Camilleri J. (2024). Leaching and cytotoxicity of bismuth oxide in ProRoot MTA—A laboratory investigation. Int. Endod. J..

[6] Parirokh M., Torabinejad M. (2010). Mineral trioxide aggregate: A comprehensive literature review—Part III: Clinical applications, drawbacks, and mechanism of action. J. Endod..

[7] Pinheiro L.S., Kopper M.P., Quintana R.M., Scarparo R.K., Grecca F.S. (2021). Does MTA provide a more favourable histological response than other materials in the repair of furcal perforations? A systematic review. Int. Endod. J..

[8] Karunakaran H., Krithikadatta J., Doble M. (2024). Local and systemic adverse effects of nanoparticles incorporated in dental materials—A critical review. Saudi Dent. J..

[9] Khalil W.A., Eid N.F. (2013). Biocompatibility of BioAggregate and mineral trioxide aggregate on the liver and kidney. Int. Endod. J..

[10] Garcia L.D.F.R., Huck C., Magalhães F.A.C., Souza P.C., Souza Costa C.A.D. (2017). Systemic effect of mineral aggregate-based cements: Histopathological analysis in rats. J. Appl. Oral Sci..

[11] Antonijević D., Despotović A., Biočanin V., Milošević M., Trišić D., Lazović V., Zogović N., Milašin J., Ilić D. (2021). Influence of the addition of different radiopacifiers and bioactive nano-hydroxyapatite on physicochemical and biological properties of calcium silicate based endodontic ceramic. Ceram. Int..

[12] Queiroz M.B., Torres F.F.E., Rodrigues E.M., Viola K.S., Martelo R.B., Chavez-Andrade G., Guerreiro-Tanomaru J.M., Tanomaru-Filho M. (2021). Physicochemical, biological, and antibacterial evaluation of tricalcium silicate based reparative cements with different radiopacifiers. Dent. Mater. J..

[13] Ilić V., Antonijević D., Petrović B., Biočanin V., Jović Orsini N., Potočnik J., Milošević M., Trajković I., Paraš S., Jokanović V. (2025). Physicochemical and biological properties of bioactive calcium aluminate dental cement enriched with zirconium dioxide. Ceram. Int..

[14] Parreira R.M., Andrade T.L., Luz A.P., Pandolfelli V.C., Oliveira I.R. (2016). Calcium aluminate cement-based compositions for biomaterial applications. Ceram. Int..

[15] Čolović B., Janković O., Živković S., Žižak Ž., Besu Žižak I., Jokanović V. (2019). A new endodontic mixture based on calcium aluminate cement obtained by hydrothermal synthesis. Ceram. Int..

[16] Primus C., Gutmann J.L., Tay F.R., Fuks A.B. (2022). Calcium silicate and calcium aluminate cements for dentistry reviewed. J. Am. Ceram. Soc..

[17] Mohammadi H., Nilforoushan M.R., Tayebi M. (2020). Effect of nanosilica addition on bioactivity and in vivo properties of calcium aluminate cement. Ceram. Int..

[18] Cetkovic D., Zhiyu C., Vukovic N., Vukovic Z., Lei H., Biocanin V., Huang X., Jokanovic V., Antonijevic D., Dozic A. (2024). The influence of different radiopacifying agents on hermetical sealing ability of calcium silicate and calcium aluminate dental cements. Sci. Sinter..

[19] Dožić A., Ćetković D., Despotović A., Janjetović K., Zogović N., Antonijević Đ. (2022). Surface morphology, compressive strength and biocompatibility of calcium aluminate dental cement. Proceedings of the International Conference on Fundamental and Applied Aspects of Physical Chemistry.

[20] Paraš S., Janković O., Trišić D., Čolović B., Mitrović-Ajtić O., Dekić R., Soldatović I., Živković Sandić M., Živković S., Jokanović V. (2019). Influence of nanostructured calcium aluminate and calcium silicate on the liver: Histological and unbiased stereological analysis. Int. Endod. J..

[21] Delfino M.M., Jampani J.L.d.A., Lopes C.S., Guerreiro-Tanomaru J.M., Tanomaru-Filho M., Sasso-Cerri E., Cerri P.S. (2021). Comparison of Bio-C Pulpo and MTA Repair HP with White MTA: Effect on liver parameters and evaluation of biocompatibility and bioactivity in rats. Int. Endod. J..

[22] Tabarsi B., Parirokh M., Eghbal M.J., Haghdoost A.A., Torabzadeh H., Asgary S. (2010). A comparative study of dental pulp response to several pulpotomy agents. Int. Endod. J..

[23] Janković O., Arbutina R., Adamović T., Đukić I., Kapetanović J., Lovrić J., Vukoje K. (2024). Histopathological pulp response of teeth capped with calcium aluminate cement and biodentine: Experimental study on rodents. Veterinaria.

[24] (2022). Biological Evaluation of Medical Devices. Part 2: Animal Welfare Requirements.

[25] Cintra L.T., Bernabé F., de Moraes I.G., Gomes-Filho J.E., Okamoto T., Consolaro A., Pinheiro T.N. (2010). Evaluation of subcutaneous and alveolar implantation surgical sites in the study of the biological properties of root-end filling endodontic materials. J. Appl. Oral Sci..

[26] (2018). Dentistry—Evaluation of Biocompatibility of Medical Devices Used in Dentistry.

[27] Aboghazleh R., Boyajian S.D., Atiyat A., Udwan M., Al-Helalat M., Al-Rashaideh R. (2024). Rodent brain extraction and dissection: A comprehensive approach. MethodsX.

[28] Smith D.G., Schenk M.P. (2001). A Dissection Guide & Atlas to the Rat.

[29] Junatas K.L., Tonar Z., Kubíková T., Liška V., Pálek R., Mik P., Králíčková M., Witter K. (2017). Stereological analysis of size and density of hepatocytes in the porcine liver. J. Anat..

[30] Knodell R.G., Ishak K.G., Black W.C., Chen T.S., Craig R., Kaplowitz N., Kiernan T.W., Wollman J. (1981). Formulation and application of a numerical scoring system for assessing histological activity in asymptomatic chronic active hepatitis. Hepatology.

[31] Perera T., Ranasinghe S., Alles N., Waduge R. (2018). Effect of fluoride on major organs with the different time of exposure in rats. Environ. Health Prev. Med..

[32] Arjmand A., Tsipouras M.G., Tzallas A.T., Forlano R., Manousou P., Giannakeas N. (2020). Quantification of liver fibrosis-A comparative study. Appl. Sci..

[33] Toprak T., Sekerci C.A., Aydın H.R., Ramazanoglu M.A., Arslan F.D., Basok B.I., Kucuk H., Kocakgol H., Aksoy H.Z., Asci S.S. (2020). Protective effect of chlorogenic acid on renal ischemia/reperfusion injury in rats. Arch. Ital. Urol. Androl..

[34] Afifi O.K., Embaby A.S. (2016). Histological study on the protective role of ascorbic acid on cadmium-induced cerebral cortical neurotoxicity in adult male albino rats. J. Microsc. Ultrastruct..

[35] Kaid F., Alabsi A.M., Alafifi N., Ali-Saeed R., Ameen Al-Koshab M., Ramanathan A., Ali A.M. (2019). Histological, biochemical, and hematological effects of Goniothalamin on selective internal organs of male Sprague-Dawley rats. J. Toxicol..

[36] Abd-Elhakim Y.M., El Sharkawy N.I., Gharib H.S.A., Hassan M.A., Metwally M.M.M., Elbohi K.M., Hassan B.A., Mohammed A.T. (2023). Neurobehavioral responses and toxic brain reactions of juvenile rats exposed to Iprodione and Chlorpyrifos, alone and in a mixture. Toxics.

[37] Shelly S., Zaltsman S.L., Ben-Gal O., Dayan A., Ganmore I., Shemesh C., Atrakchi D., Garra S., Ravid O., Rand D. (2021). Potential neurotoxicity of titanium implants: Prospective, in-vivo and in-vitro study. Biomaterials.

[38] Souza C.C., Barreto T.O., da Silva S.M., Pinto A.W.J., Figueiredo M.M., Ferreira Rocha O.G., Cangussú S., Tafuri W.L. (2014). A potential link among antioxidant enzymes, histopathology and trace elements in canine visceral leishmaniasis. Int. J. Exp. Pathol..

[39] Kareem D.A., Ali S.A., Sadoon A.H., Al-Mousawi Z.A.H., Alallawee M.H.A. (2022). Anatomical and histological alterations of the spleen in rat, *Rattus norvegicus* exposed to mercury. Iran. J. Ichthyol..

[40] (2018). Biological Evaluation of Medical Devices. Part 1: Evaluation and Testing Within a Risk Management Process.

[41] Upton R.N., Doolette D.J. (1999). Kinetic aspects of drug disposition in the lungs. Clin. Exp. Pharmacol. Physiol..

[42] Astudillo-Ortiz E., Babo S., Gonçalves A.I., Gomes M.E. (2025). Magnetic cell-homing strategy for autologous dental pulp regeneration as an alternative in necrotic teeth: A proof-of-concept study. Eur. Cell Mater..

[43] Torabinejad M., Corr R., Buhrley M., Wright K., Shabahang S. (2011). An animal model to study regenerative endodontics. J. Endod..

[44] Bryda E.C. (2013). The mighty mouse: The impact of rodents on advances in biomedical research. Mo. Med..

[45] (2017). Biological Evaluation of Medical Devices. Part 11: Tests for Systemic Toxicity.

[46] Cantiga-Silva C., Estrela C., Segura-Egea J.J., Azevedo J.P., de Oliveira H.C., Cardoso C.B.M., Pinheiro T.N., Ervolino E., Sivieri-Araújo G., Cintra L.T.A. (2021). Inflammatory profile of apical periodontitis associated with liver fibrosis in rats: Histological and immunohistochemical analysis. Int. Endod. J..

[47] Rinwa P., Eriksson M., Cotgreave I., Bäckberg M. (2024). 3R-refinement principles: Elevating rodent well-being and research quality. Lab. Anim. Res..

[48] Atas O., Bilge K., Yıldız S., Dundar S., Calik I., Gezer Atas A., Bozoglan A. (2023). Systemic effect of calcium silicate-based cements with different radiopacifiers—Histopathological analysis in rats. PeerJ.

[49] Paraš S., Trišić D., Mitrović Ajtić O., Antonijević Đ., Čolović B., Drobne D., Jokanović V. (2021). Biocompatibility study of a new dental cement based on hydroxyapatite and calcium silicates: Focus on liver, kidney, and spleen tissue effects. Int. J. Mol. Sci..

[50] Sengupta P. (2013). The laboratory rat: Relating its age with human’s. Int. J. Prev. Med..

[51] Torneck C.D. (1966). Reaction of rat connective tissue to polyethylene tube implants. Oral Surg. Oral Med. Oral Pathol..

[52] (2016). Biological Evaluation of Medical Devices.

[53] Toledano-Serrabona J., Camps-Font O., de Moraes D.P., Corte-Rodríguez M., Montes-Bayón M., Valmaseda-Castellón E., Gay-Escoda C., Sánchez-Garcés M.Á. (2023). Ion release and local effects of titanium metal particles from dental implants: An experimental study in rats. J. Periodontol..

[54] Bramante C.M., Demarchi A.C.C.O., de Moraes I.G., Bernadineli N., Garcia R.B., Spångberg L.S., Duarte M.A.H. (2008). Presence of arsenic in different types of MTA and white and gray Portland cement. Oral Surg. Oral Med. Oral Pathol. Oral Radiol. Endod..

[55] Chang S.-W., Baek S.-H., Yang H.-C., Seo D.-G., Hong S.-T., Han S.-H., Lee Y., Gu Y., Kwon H.-B., Lee W. (2021). Heavy metal analysis of ortho MTA and ProRoot MTA. J. Endod..

[56] Lentini P., Zanoli L., Granata A., Signorelli S.S., Castellino P., Dell’Aquila R. (2017). Kidney and heavy metals—The role of environmental exposure (Review). Mol. Med. Rep..

[57] da Fonseca T.S., da Silva G.F., Tanomaru-Filho M., Sasso-Cerri E., Guerreiro-Tanomaru J.M., Cerri S. (2016). In vivo evaluation of the inflammatory response and IL-6 immunoexpression promoted by Biodentine and MTA Angelus. Int. Endod. J..

[58] Silva E.C.A., Tanomaru-Filho M., Silva G.F., Lopes C.S., Cerri S., Guerreiro-Tanomaru J.M. (2021). Evaluation of the biological properties of two experimental calcium silicate sealers: An in vivo study in rats. Int. Endod. J..

[59] Zulfiqar N.S. (2014). Pyramidal layer thinning, shrunken neurons and deep vacuolation in hippocampus due to the organic lead induced toxicity. Int. J. Anat. Res..

[60] Diethorn E.J., Gould E. (2023). Development of the hippocampal CA2 region and the emergence of social recognition. Dev. Neurobiol..

[61] Jordan W.H., Young J.K., Hyten M.J., Hall D.G. (2010). Preparation and Analysis of the Central Nervous System. Toxicol. Pathol..

[62] Yuan J., Lipinski M., Degterev A. (2003). Diversity in the mechanisms of neuronal cell death. Neuron.

[63] Lewis S.M., Williams A., Eisenbarth S.C. (2019). Structure and function of the immune system in the spleen. Sci. Immunol..

[64] Bende R.J., van Maldegem F., van Noesel C.J. (2009). Chronic inflammatory disease, lymphoid tissue neogenesis and extranodal marginal zone B-cell lymphomas. Haematologica.

[65] Santana M.A., de Almeida Chaves D.S., de Andrade L.A., Santos E.L., da Silva S.V. (2021). Ovariectomy and chronic stress: Effects on the morphometry of the spleen of rats. J. Morphol. Sci..

